# Performance of a Non-Invasive System for Monitoring Blood Glucose Levels Based on Near-Infrared Spectroscopy Technology (*Glucube^®^*)

**DOI:** 10.3390/s24237811

**Published:** 2024-12-06

**Authors:** Fernando Gómez-Peralta, Luis Gabriel Luque Romero, Antonio Puppo-Moreno, Jesús Riesgo

**Affiliations:** 1 Endocrinology and Nutrition Unit, Segovia General Hospital, Luis Erik Clavería Neurólogo S.N Street, 40002 Segovia, Spain; 2 Health Care Center La Algaba, 41980 La Algaba, Spain; investigacion.daljsevn.sspa@juntadeandalucia.es; 3 Investigation Unit of the Aljarafe District-Sevilla Norte, 41008 Seville, Spain; 4 Intensive Care Unit, Hospital Universitario Virgen Del Rocío, Avda. Manuel Siurot, s/n, 41013 Seville, Spain; antoniom.puppo.sspa@juntadeandalucia.es; 5 GLUCUBE, 41011 Seville, Spain; jesus@glucube.com

**Keywords:** *Glucube^®^*, diabetes, non-invasive glucose monitoring, self management, adherence

## Abstract

Background: The need for frequent blood glucose (BG) monitoring and the inconveniences associated with self-monitoring of BG (SMBG) have driven the development of non-invasive approaches. Methods: This prospective study aimed to investigate the accuracy of glucose level calculation using the near-infrared spectroscopy (NIRS) technology *Glucube^®^* system. People with Type 1 diabetes, Type 2 diabetes, prediabetes, and normal glucose metabolism were included. Over one week, individuals performed glucose measurements with the *Glucube^®^* system and capillary blood fingersticks with a standard glucometer (*Ascensia Contour^®^ Next*). To assess the impact of the improvement in dexterity, the accuracy variables were compared with the point-of-care (POC) glucometer Accu-Chek^®^ Inform II in a one-week sub-study. Results: Overall, 105 subjects (mean age 53.8 ± 13.8 years, 50.5% female) participated, resulting in 1914 paired glucose measurements between 49 and 331 mg/dL. Total mean absolute relative difference (MARD) was 20.3%, MARD for values >100 mg/dL was 18.3%, and mean absolute deviation (MAD) for values <100 mg/dL was 24.9%. A total of 97.3% of measurements fell within A+B Parkes zones, and 58.8%, 76.9%, and 88.1% within +−20%, +−30%, or +−40% error, respectively. On completion, 62 participants (59%) fulfilled the one-week prospective sub-study. In this subgroup, the total MARD was reduced between day 1 and day 8 from 22.8 to 18.3% (*p* = 0.068). The percentages within Zone A were 51.6 vs. 61.2%, Zone B 46.8 vs. 33.9%, and Zone C 1.6 vs. 4.8%, and the sum of Parkes Zones A+B was 98.4 vs. 95.2% (*p* = 0.311) for day 1 and day 8, respectively. Conclusions: *Glucube^®^* is a novel non-invasive system based on NIRS technology for monitoring blood glucose levels. Its promising capabilities support further research.

## 1. Introduction

Diabetes mellitus (DM) is considered a major public health problem because of its ongoing increase in prevalence and its impact on mortality, morbidity, and healthcare costs [1,2]. The natural progression of prediabetes, Type 1 DM (T1D), and Type 2 DM (T2D) is a slow process that often takes several decades to progress from early stages to advanced comorbid states. Unfortunately, organ damage accumulates right from the beginning, and there is limited scope for improvement in the established complications as the patient ages [3]. The current diagnosis and definition for both prediabetes and DM are mainly based on glycemic criteria [4]. Early glycemic diagnosis and monitoring are critical. Recently published data from the UK Clinical Practice Research Datalink database confirmed that at the time of the diagnosis of T2D, half of the study population had at least one vascular disease [5]. Something critical from this study is that those with prediabetes glucose data before the T2D diagnosis had 76% and 14% increased odds of retinopathy and nephropathy and 7% higher odds of the diagnosis of acute coronary syndrome at the time of DM diagnosis. The findings highlight the necessity of developing risk-reduction strategies that address the full spectrum of dysglycemia. However, the prevalence of unknown DM in adults can reach 40% of the total DM [6]. The considerable prevalence of unknown DM in Western countries can be attributed, in part, to individuals’ aversion to invasive procedures for early detection and ongoing monitoring [7,8].

The conventional finger-prick method, though accurate, is not feasible for use multiple times a day as it is painful, and test strips are expensive [9]. Non-invasive monitoring of glucose levels is a very active area of research. However, a sufficiently accurate non-invasive device to replace blood sampling still needs to be improved. Developing non-invasive sensors with accuracy similar to capillary blood glucose-monitoring devices and more affordability could change how DM is managed. Several methods not requiring any incision or implantation and, therefore, are painless and less uncomfortable, have been proposed. However, all of them are still in the testing phase.

A number of minimally invasive and non-invasive technologies for glucose detection are based on optical methods [10]. Optical spectroscopy is based on the absorption of light radiation by vibrating molecules. A molecule absorbs energy from a light beam when its vibrational frequency matches the wavelength. Therefore, glucose levels can be estimated from the change in light intensity passing through a tissue. Optical spectroscopy includes near-infrared spectroscopy (NIRS), with wavelengths ranging from 700 nm to 2500 nm, and mid-infrared spectroscopy (MIRS), ranging from 2500 nm to 10 μm. The high water absorption in the MIRS region prevents signals from penetrating more than a few micrometers into the tissue, needing the use of powerful MIRS sources such as quantum cascade lasers (QCLs) [11].

The *Glucube^®^* system is based on NIRS technology. The absorption of infrared radiation is measured by the emission of NIRS radiation of controlled intensity and the measurement of the intensity received by a photoreceiver after passing through the tissues of the patient’s finger.

This clinical study aimed to compare the performance of the non-invasive *Glucube^®^* device in monitoring and measuring blood glucose in adult patients to that of a standard blood capillary glucometer.

## 2. Materials and Methods

This study complied with the protocol, the Declaration of Helsinki—Ethical Principles for Medical Research Involving Human Participants, ISO 14155:2020 (Clinical Investigation of Medical Devices for Human Participants—Good Clinical Practice) [12], and applicable regulatory requirements. The protocol, informed consent form (ICF) combined with a participant information sheet, and all appropriate study-related documents were reviewed and approved on 2 February 2024 by the Ethics on Investigation Committee (Comité de Ética de la Investigación Provincial de Sevilla, DIA-2021-01) and by the Spanish Agency for Medicines and Sanitary Products, AEMPS). All the participants provided informed consent.

### 2.1. Description of the Investigational Device (Glucube^®^)

The *Glucube^®^* system consists of software and hardware elements (Supplementary Figure S1). The hardware element is a portable device that utilizes NIRS technology to take measurements from the various infrared beams that pass through the users’ skin on the finger. The measurement module of the device consists of an infrared LED with different wavelengths ranging from 700 nm to 2500 nm. Employing optical spectroscopy, the variation in light intensity after energy absorption by glucose molecules is measured. These measurements are sent via Bluetooth to the *Glucube^®^* app installed on the user’s smartphone. The app feeds the algorithm in the cloud, which processes the signal to obtain the blood glucose measurement. The algorithm considers the variation in the intensity of the different infrared light beams in addition to inputs about the user, such as height, weight, age, and sex. The algorithm analyzes all these data to provide a refined and accurate measure of blood glucose levels. The algorithm returns the blood glucose value to the *Glucube^®^* app, which displays the measurement. Finally, the *Glucube^®^* web platform facilitates the monitoring and management of diabetes, sharing glucose data and other health-related information with the caregivers.

Ambient light can affect non-invasive optical technologies. The *Glucube^®^* system has several measures to prevent interference. Its design isolates the measuring area from ambient light when a finger is placed inside the device. Additionally, the system can detect if ambient light influences the results.

The impact of sweat on NIRS depends on the method used: reflection-based NIRS vs. diffraction-based NIRS. The *Glucube^®^* system, which is diffraction-based NIRS, involves deeper tissue interactions, making it less sensitive to superficial factors like sweat. Nevertheless, patients were advised to keep their hands dry when measuring.

### 2.2. Description of the Reference Glucometer Device (Ascensia Contour^®^ Next)

The reference device used during this clinical study is a standard capillary blood glucometer, *Ascensia Contour^®^ Next* (Ascensia Diabetes Care Holding AG, 4052 Basel, Switzerland) (ACN). It works by applying a sample of blood obtained by a finger prick to a test strip inserted into the glucometer. The device then uses a chemical reaction (an enzymatic method based on glucose dehydrogenase, flavin adenine dinucleotide) in their test strips to determine the glucose concentration in the sample. When compared with five other blood glucose glucometers, ACN demonstrated the lowest mean deviation from the reference value across multiple glucose ranges [13].

### 2.3. Population

Participants were required to be ≥18 years old and have a smartphone with an internet connection that allows the installation of the *Glucube^®^* application. Participants were excluded if they presented anatomical conditions or comorbidities that could limit their ability to partake in clinical research, such as being affected by an acute active infectious disease, having a condition that impacts finger blood circulation, participants with nail polish or any false nail, or those who did not have sufficient manual dexterity to perform required tests independently.

### 2.4. Study Design

This study was a prospective, single-center, non-interventional, open-label clinical investigation. Two visits were scheduled, with 8 + 2 days between them. Visit 1 (day 1) involved screening and participant inclusion. Participant informed consent was obtained, and demographics, height and weight, medical history, and medication information were collected. Participants were introduced to the *Glucube^®^* device and the standard glucometer ACN.

On days 2 through 7, participants took four paired glucose measurements with the investigational device and the standard glucometer ACN. Measurements were taken in the morning fasting state (fasting), one hour before lunch (pre-prandial), two hours after lunch (post-prandial), and one hour before sleep (nocturnal).

Participants reported any device deficiency or adverse events. All subjects received follow-up as per the standard of care.

### 2.5. Primary Performance Endpoint

This study aimed to measure the *Glucube^®^* device’s accuracy in measuring blood glucose in adult patients compared to the standard blood capillary glucometer. International guidelines for blood glucose measurement devices explicitly state that they are not applicable to non-invasive technology [12,14]. Furthermore, scientific societies in the United States and Europe have proposed various accuracy standards. Nevertheless, the present study utilized both statistical estimations designed for minimally invasive or invasive methods.

The mean absolute percentage bias was calculated using the mean absolute relative difference (total MARD). The MARD was also calculated for values > 100 mg/dL (MARD) using the relative error, and the mean absolute deviation (MAD) was computed for the values < 100 mg/dL. In addition, the ISO15197:2015-recommended consensus error grid (CEG) analysis, according to Parkes et al. [12,15], was also performed. The pairs of measurements were represented in the CEG, which evaluated the clinical significance of the inaccuracy in blood glucose measurement. The ratio of measurement pairs from the investigational device to the reference device was calculated as the number of valid pairs in each zone divided by the total valid pairs in any zone. Zone A is defined in the Parkes error grid as “clinically accurate measurements with no effect on clinical action”, and Zone B as the zone of “altered clinical action with little or no effect on clinical outcome”. In Zone C, errors may lead to unnecessary treatments, while Zone D involves delays in appropriate treatment, which could be harmful. Lastly, Zone E refers to severe errors that could result in life-threatening decisions.

The percentage of measurements with error +−20%, +−30%, and +−40% was calculated separately for measurements with a reference value > or <100 mg/dL.

### 2.6. Secondary Performance Analysis

A one-week prospective sub-study was included to assess the impact of improving the user’s dexterity with the device. The accuracy variables on day 1 and day 8 were compared to track the device’s performance over time.

### 2.7. Statistical Analyses

We carried out calculations relating to sample size to ensure we recruited an appropriate number of participants. Based on the primary study, a Clarke error grid analysis was performed, giving a 99.26% result of measured investigational versus reference data-paired observations that were within regions A and B. In this study, improvements in the investigational device and less sample heterogeneity led us to think of 99.35% of this result. Assuming that the exploratory graph analysis follows a binomial distribution, a sample size of 1500 (60 participants × 25 pairs of measurements across six days) of investigational versus the reference device was estimated to provide a success probability of 95%. We also adjusted the account for study dropouts (20%), incomplete or not performed measurements (20%), or measurements that fell between the range of 70–180 mg/dL (10%). This, therefore, required a recruitment of 105 patients, each with 25 valid measurement pairs.

Analyses were carried out when the total pre-planned sample size was reached, and the database was discrepancy-free. Only observed data were analyzed.

### 2.8. Safety

Adverse events were collected, including, but not limited to, redness, burns, pain, or other complications such as bleeding or local infection.

## 3. Results

### 3.1. Subjects

Of the 128 participants with signed informed consent, 111 completed this study. No withdrawals due to adverse events were reported. The final evaluable subjects’ number was 105. The mean age of the subjects was 53.8 + 13.8 years, and 50.5% were female. In all, 2 (1.9%) subjects had T1D, 35 (33.3%) had T2D, 2 (1.9%) had prediabetes, and 66 (62.9%) were normoglycemic subjects.

The study procedures were well tolerated. No general adverse events or device-related adverse events were reported. The participant’s demographics and clinical characteristics data are described in Table 1.

### 3.2. Glucose Dataset

In this study, 1918 records were collected, of which 1914 were valid measurements.

**Figure 1:**
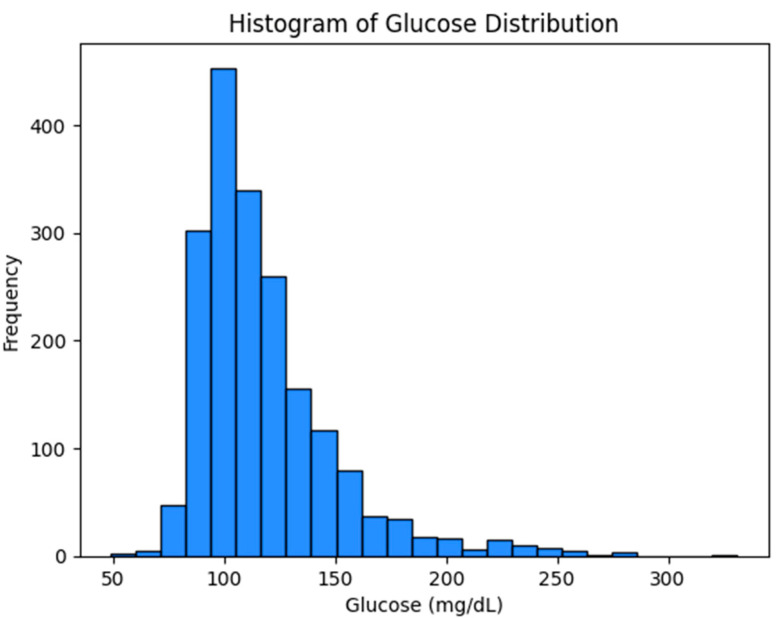
Histogram of glucose data distribution.

The distribution of the glucose reference values obtained for the clinical analysis is represented in Figure 1.

### 3.3. Primary Performance Analysis

Total MARD was 20.3%, MARD for values > 100 mg/dL: 18.3%, MAD for values < 100 mg/dL: 24.9%.

A total of 97.3% of measurements fell within A + B Parkes zones. The percentage of results in Zone A of the Parkes error grid was 62.0%, Zone B 35.3%, Zone C 2.6%, Zone D 0.05%, and Zone E 0%.

**Figure 2:**
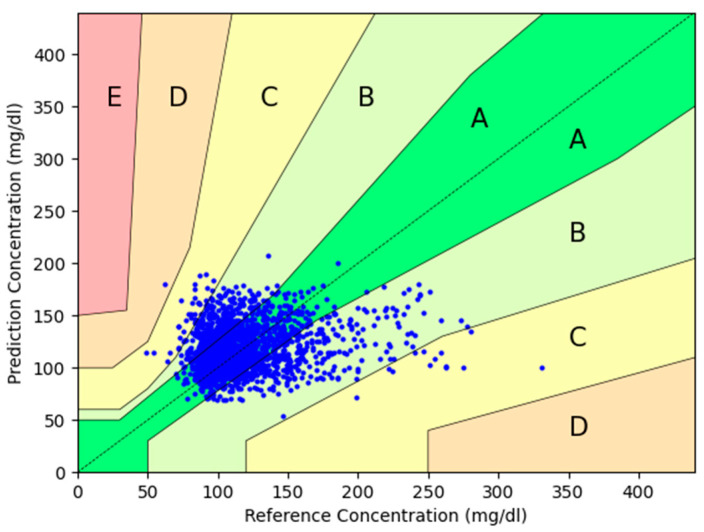
Parkes error grid of the total glucose paired measurements. Blue dots represent the pairs of glucose measurements from the investigational device compared to the reference device. Zone A is defined in the Parkes error grid as “clinically accurate measurements with no effect on clinical action”, and Zone B as the zone of “altered clinical action with little or no effect on clinical outcome”. In Zone C, errors may lead to unnecessary treatments, while Zone D involves delays in appropriate treatment, which could be harmful. Lastly, Zone E refers to severe errors that could result in life-threatening decisions.

The plot is shown in Figure 2, including the percentage of measurements in each zone.

For measurements with a reference value < 100 mg/dL, the percentage of measurements with error ±20 mg/dL, ±30 mg/dL, and ±40 mg/dL was 52.0%, 68.6%, and 80.2%, respectively. For measurements with a reference value ≥ 100 mg/dL, the percentage of measurements with error ±20%, ±30%, and ±40% was 61.7%, 80.6%, and 91.6%, respectively.

### 3.4. Secondary Performance Analysis

A total of 62 participants (59%) completed the one-week prospective sub-study. In this subgroup, the total MARD was reduced between day 1 and day 8 from 22.8 to 18.3% (*p* = 0.068). MARD for values > 100 mg/dL on day 1 and day 8 were 22.2% vs. 18.1% (*p* = 0.310). Mean absolute deviation (MAD) for values ≤ 100 mg/dL was 24.1 mg/dL vs. 19.5 mg/dL (*p* = 0.671).

The percentages within Zone A were 51.6 vs. 61.2%; Zone B 46.8 vs. 33.9%; Zone C 1.6 vs. 4.8%, and the sum of Parkes Zones A+B was 98.4 vs. 95.2% (*p* = 0.311) for day 1 and day 8, respectively.

The percentages of measurements with error within +−20%, +−30%, and +−40% were 48.4%, 64.5%, and 88.7% on day 1 and 61.3%, 77.4%, and 88.71% on day 8.

**Figure 3:**
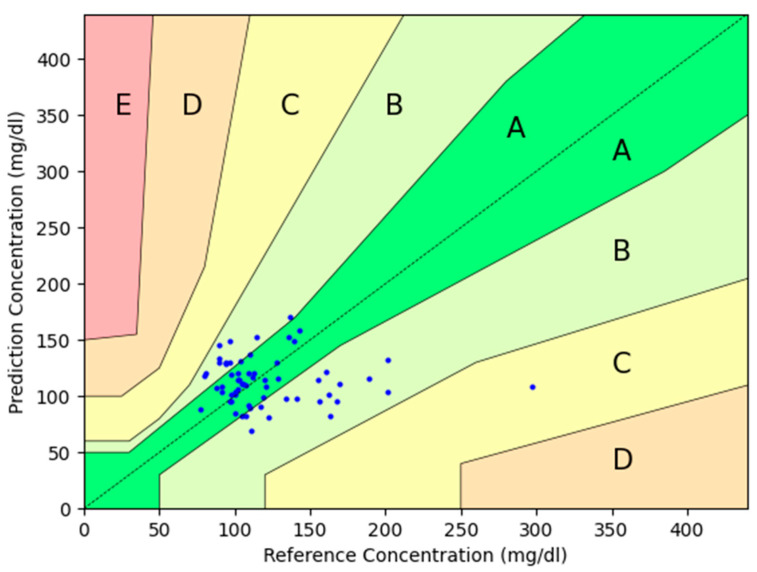
Parkes error grid sub-study glucose measures on the first day of use. Blue dots represent the pairs of glucose measurements from the investigational device compared to the reference device. Zone A is defined in the Parkes error grid as “clinically accurate measurements with no effect on clinical action”, and Zone B as the zone of “altered clinical action with little or no effect on clinical outcome”. In Zone C, errors may lead to unnecessary treatments, while Zone D involves delays in appropriate treatment, which could be harmful. Lastly, Zone E refers to severe errors that could result in life-threatening decisions.

**Figure 4:**
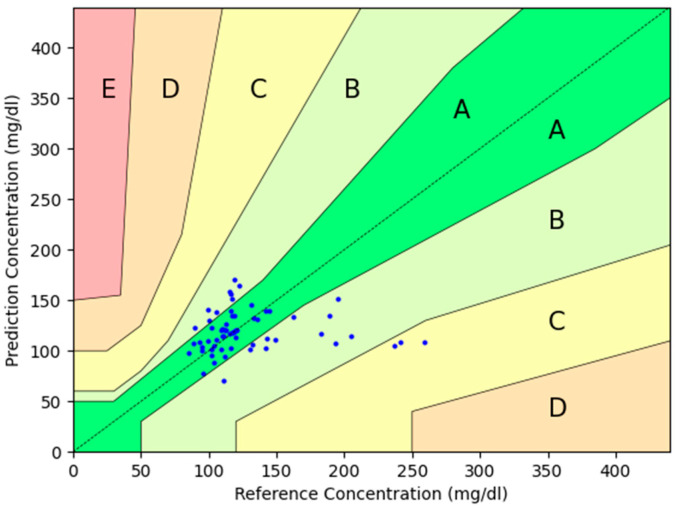
Parkes error grid sub-study glucose measures after a week of use. Blue dots represent the pairs of glucose measurements from the investigational device compared to the reference device. Zone A is defined in the Parkes error grid as “clinically accurate measurements with no effect on clinical action”, and Zone B as the zone of “altered clinical action with little or no effect on clinical outcome”. In Zone C, errors may lead to unnecessary treatments, while Zone D involves delays in appropriate treatment, which could be harmful. Lastly, Zone E refers to severe errors that could result in life-threatening decisions.

The plot is shown in Figure 3 and Figure 4, including the percentage of measurements in each zone.

## 4. Discussion

This study testing the *Glucube^®^* system accuracy showed that total MARD was 20.3%, MARD for values > 100 mg/dL was 18.3%, and MAD for values < 100 mg/dL was 24.9 mg/dL. A total of 97.3% of measurements fell within A+B Parkes zones.

Recent studies have shown that NIRS technology can be useful to estimate blood glucose levels [16,17,18,19,20,21,22,23]. It is important to note that the current U.S. Food and Drug Administration (FDA) guidance document for self-monitoring blood glucose test systems (SMBGs) is limited to those that require the removal of a blood sample from a fingertip or another anatomical site [14]. Additionally, the International Organization for Standardization (ISO) standards do not apply as well to the ‘Non-invasive’ Glucose Monitoring for Diabetes (NIO-GM) devices [12]. The Parkes error grid analysis showed that 97.3% of measurements fell within Zones A and B, and 2.6% fell into Zone C. Although the device meets the accuracy standards, the goal is to have 0.0% in Zone C in the future. Specific guidance for non-invasive blood glucose measuring technology should be developed.

Improving the accuracy of results can be achieved through the continuous use and optimization of the measurement technique. However, the small sample size in the follow-up sub-study limited our ability to achieve statistically significant results. For instance, previous studies that employed a contact pressure monitoring system using a pressure transducer demonstrated a clear enhancement in the accuracy of a non-invasive MIRS optical glucose measurement device [24]. Continuous work should be carried out to analyze data in the real world, to improve the measurement technique, and to compare them with reference blood glucose measurements to optimize the hardware and software.

Individuals with darker skin tones, such as those of African descent, have more melanin in their epidermis, which is contained in larger, more widely distributed melanosomes compared to lighter-skinned populations, such as those of European descent [25]. However, the distribution of melanin in areas like the fingertips or palms is typically less dense in all populations due to the unique properties of thick, glabrous skin in these regions [26]. Our data on subjects with darker skin pigmentation have shown that their measurements are consistent with those of volunteers without pigmentation. However, to validate these findings further, it will be necessary to include skin pigmentation analysis in future research.

The *Glucube^®^* device was designed to offer a painless and non-invasive approach to measuring BG levels, intended to complement, not replace, the standard capillary glucometer. The product meets the defined purpose of use and allows non-invasive measurements to improve glucose monitoring over time.

## 5. Conclusions

The *Glucube^®^* system provides BG real-time readings and aids in detecting glucose level excursions outside the desired range. It is intended for everyday use to detect long-term trends and guide future patient management while also reducing the number of daily finger pricks. The promising capabilities the *Glucube^®^* system offers for BG monitoring support further research.

## Figures and Tables

**Table 1:** Participant demographics and clinical characteristics.

		Total (n = 105)
Gender (n, %)	Male	52, 49.5
	Female	53, 50.5
Race (n, %)	Caucasian Hispanic Latino	103, 98.1 1, 095
	Other	1, 0.95
Education level (n, %)	Less than primary school	5, 4.8
	Completed primary school	44, 41.9
	Completed secondary school	30, 28.6
	University degree	25, 23.8
	Post-graduate degree	1, 0.95
Participant distribution (n, %)	Type 1 diabetes	2, 1.9
	Type 2 diabetes	35, 33.3
	Prediabetes	2, 1.9
	Non-diabetic	66, 62.9
Age (years)	SD	13.8
	Mean	53.8
	Min	19
	Max	76
Height (m)	SD	0.099
	Mean	1.66
	Min	1.44
	Max	1.90
Weight (kg)	SD	17.1
	Mean	83.5
	Min	45.6
	Max	142.7
BMI (kg/m^2^)	SD	6.1
	Mean	30.5
	Min	16.6
	Max	57.9
HbA1c (%)	N	95
	SD	0.97
	Mean	6.06
	Min Max	4.9 10.7

BMI, body mass index; HbA1c, glycated hemoglobin A1c; N, number of participants; SD, standard deviation.

## Data Availability

The original data presented in this study are openly available in GLUCUBE RESEARCH & DEVELOPMENT at https://www.glucube.com/research-development (accessed on 2 November 2024).

## Supplementary Materials

The following supporting information can be downloaded at: https://www.mdpi.com/article/10.3390/s24237811/s1, Supplementary Figure S1: *Glucube^®^* architecture system.
