# Supplementary figures and images for: Performance of a Non-Invasive System for Monitoring Blood Glucose Levels Based on Near-Infrared Spectroscopy Technology (Glucube®)
Source: Sensors (Basel). 2024 Dec 6;24(23):7811. doi: 10.3390/s24237811 (PMC11645037; doi:10.3390/s24237811)

Supplementary Figure S1. *Glucube*<sup>®</sup> architecture system

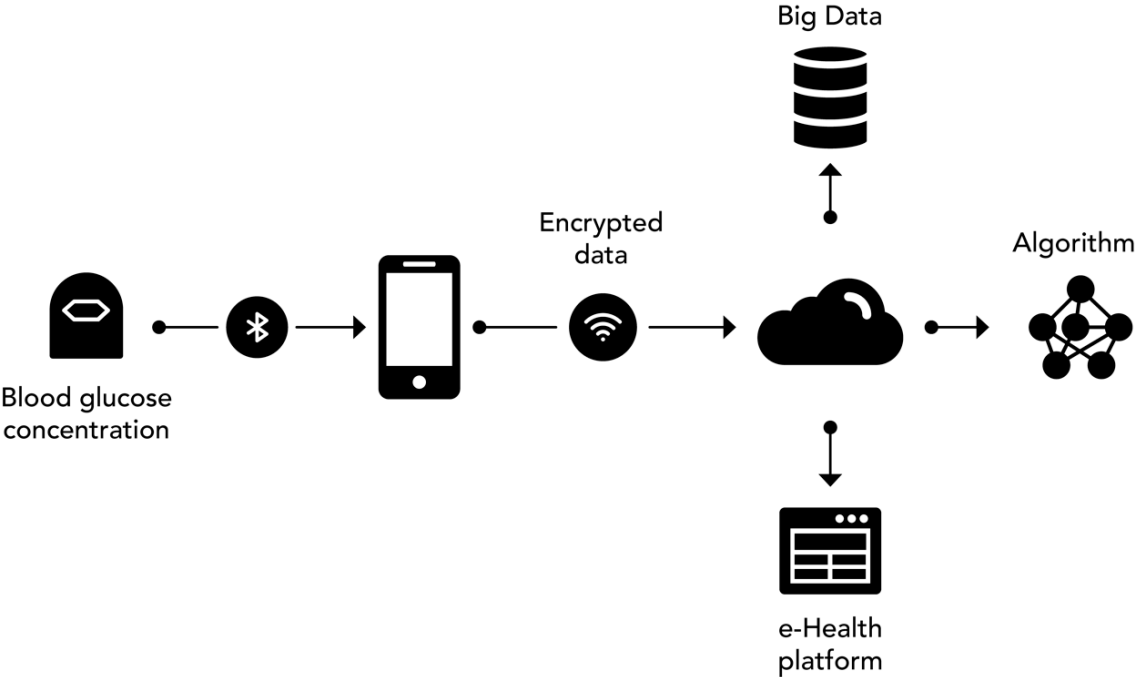

Supplement: Supplementary file 1 [file sensors-24-07811-s001.zip › sensors-3319917-supplementary.pdf]
